# Supplementary material for: A targeted sequencing extension for transcript genotyping in single-cell transcriptomics
Source: Life Sci Alliance. 2023 Sep 11;6(11):e202301971. doi: 10.26508/lsa.202301971 (PMC10494938; doi:10.26508/lsa.202301971)
Supplement: Supplemental Data 1. — Deciding on extra correction based on Hamming distance. [file LSA-2023-01971_Supplemental_Data_1.docx]

***Note S1 – Deciding on extra correction based on Hamming distance***

We suggest the following stepwise approach to decide on whether to include an extra correction based on Hamming distance (see also **Supplementary note 4** for code to generate the datasets and plots). Depending on the exact application, one may opt for being more or less strict in the used cut-offs.

*Step 1 - Distribution of reads per tagging sequence*: Unequal amplification may be clearly visible from plotting the distribution of reads per tagging sequence (see **Figure 2**, **Supplementary figures 2-5**). An exponential decrease in number of tagging sequences with increasing number of reads per tagging sequence is ideal. An excessive number of tagging sequences supported by only one read in combination with the presence of outliers with a high number of reads per tagging sequence indicates that an extra correction may be required.

The proportion of tagging sequences supported by only one read offers a quantitative measure to help with the decision. We observed up to 40% of tagging sequences supported by one read in datasets that seemed to not need extra correction, and more than 70% in datasets that benefited from the extra correction.


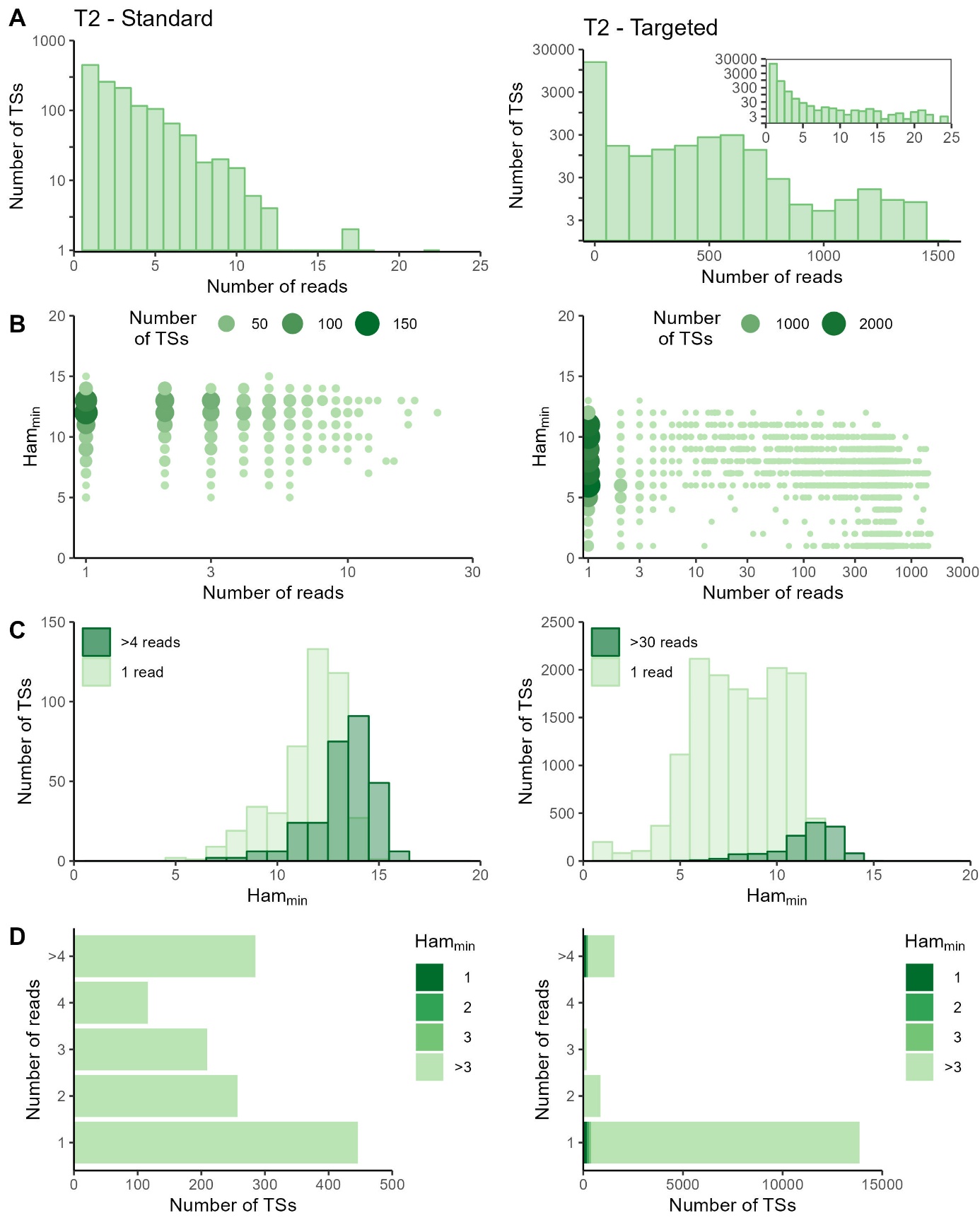
Example 1 – TREX1 T2 standard: The plot on the right shows that the distribution is close to an exponential decrease, without an excess number of tagging sequences supported by only one read and no tagging sequences with an extremely high number of reads supporting them. Only 33.97% of tagging sequences are supported by one read. Therefore, additional correction based on Hamming distances may not be necessary.


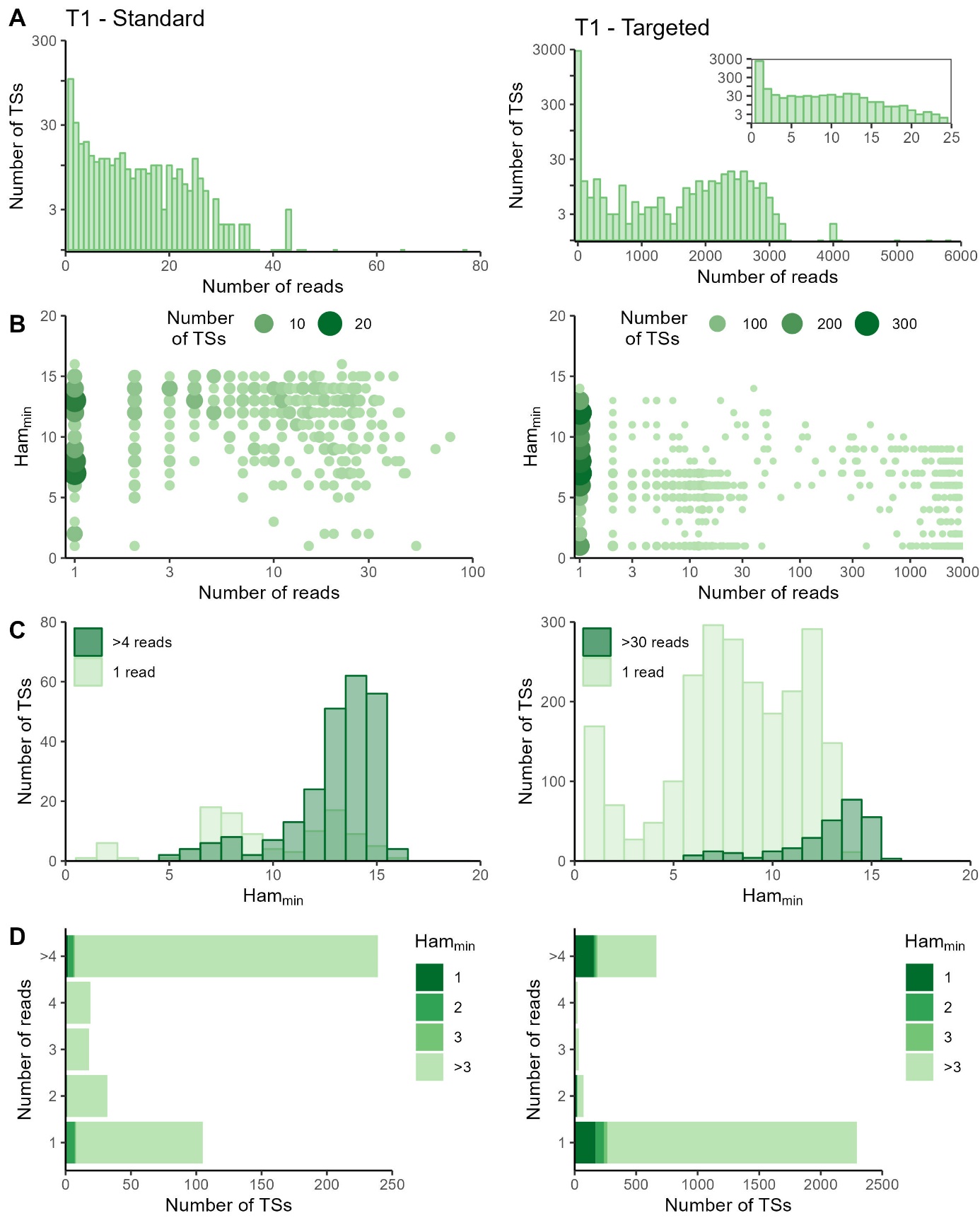
Example 2 - TREX1 T1 targeted: The plot on the right does not look like an exponential decrease. Instead, an excess number of tagging sequences are supported by only one read (74.28%), and some tagging sequences have an extremely high number of reads (>4000). Therefore, additional correction based on Hamming distances may be useful, depending on the application.

*Step 2 – Calculating minimal Hamming distances (Ham_min_) between tagging sequences*: If the previous step is insufficient to make the decision on whether to apply a correction or not, calculating the minimal Hamming distances between tagging sequences may help. As it is computationally intensive, we suggest it only if the first one is inconclusive.

Although for the given examples, step 1 suffices to decide on extra correction, we use the same examples to demonstrate step 2.


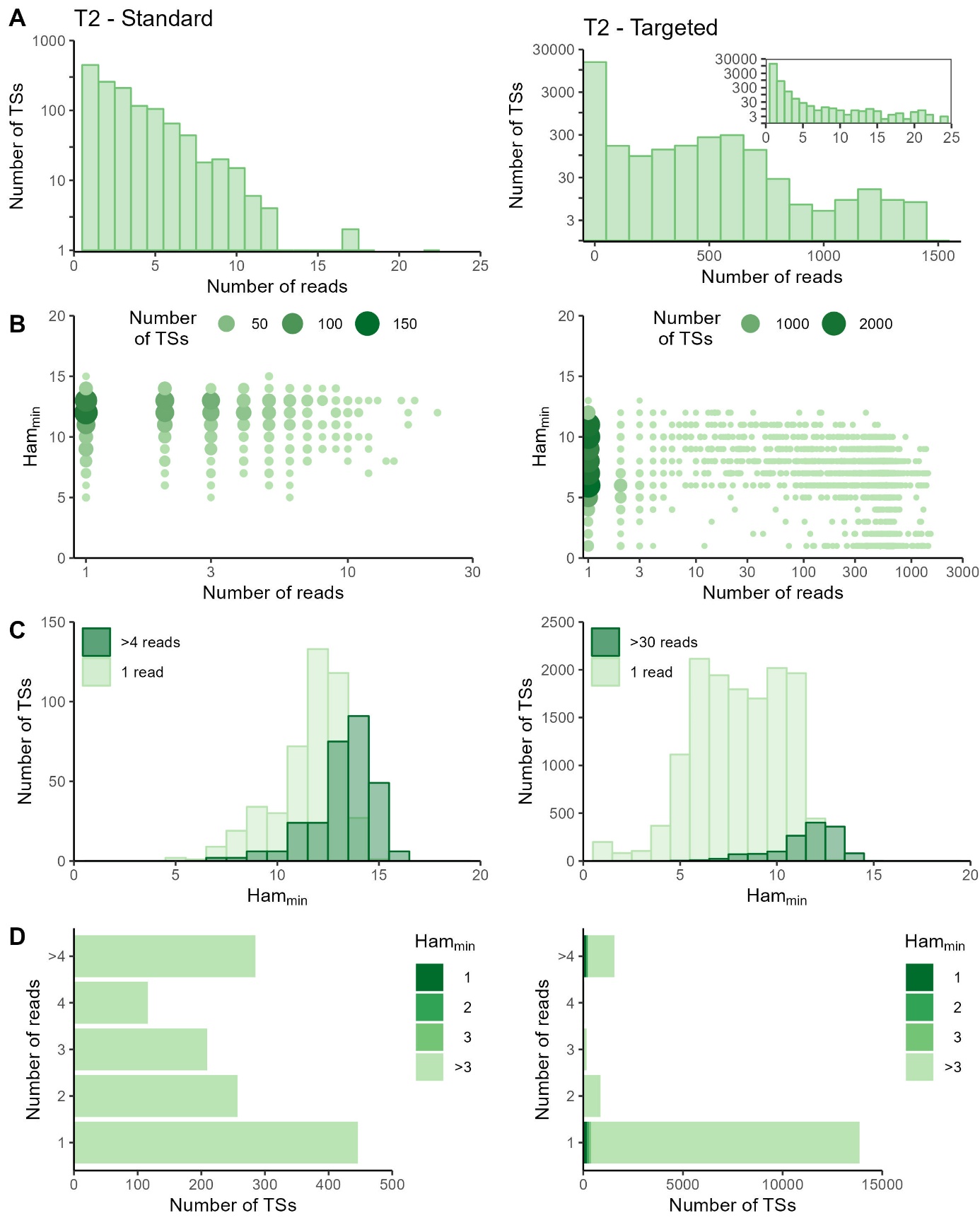
Example 1 – TREX1 T2 standard: The upper plot on the right shows that the minimal Hamming distance between tagging sequences within this dataset is at least 5, indicating that the tagging sequences are very likely capturing independent transcripts.


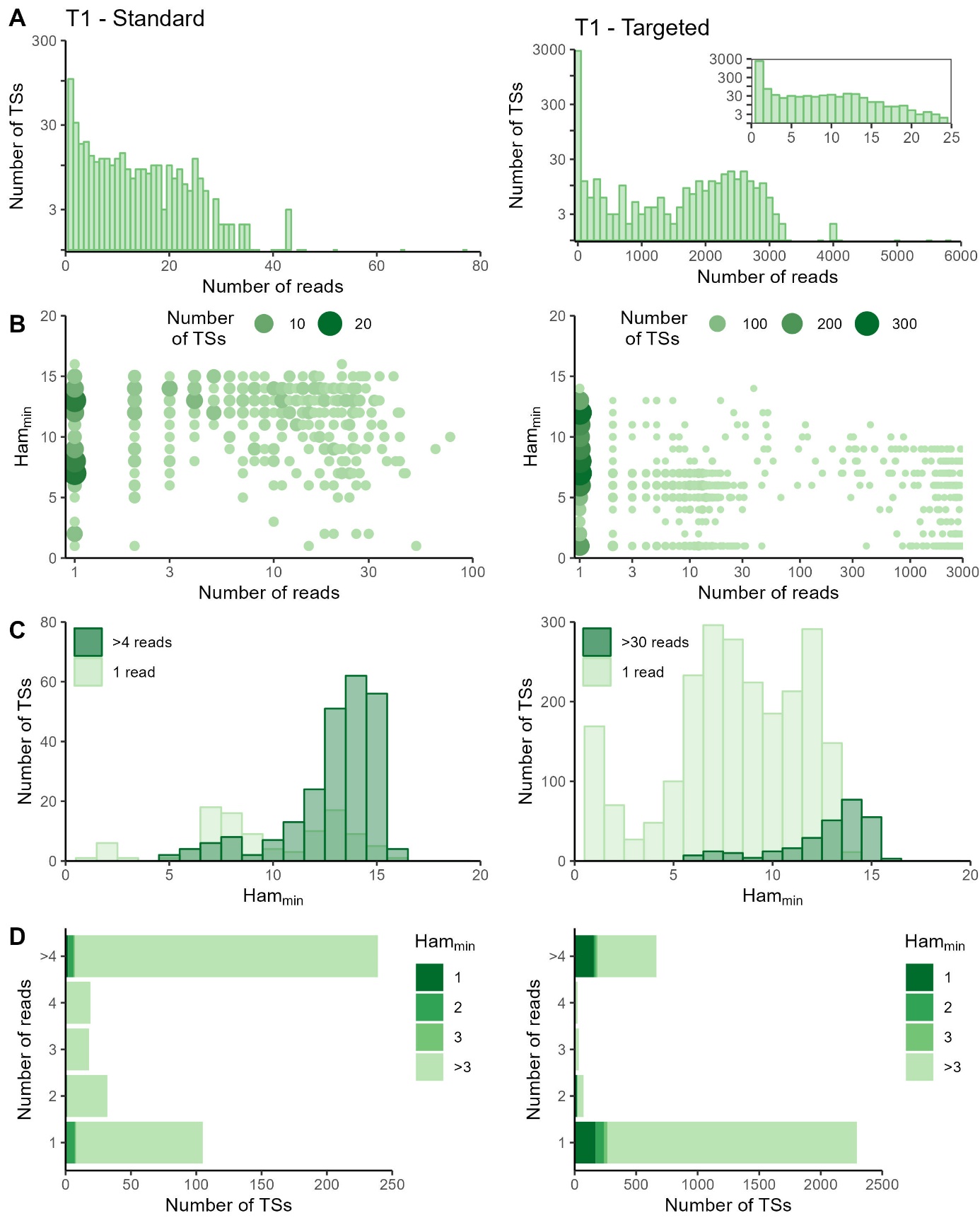
Example 2 – TREX1 T1 targeted: The lower plot on the right indicates that for a substantial part of tagging sequences, the minimal Hamming distance is ≤3. While this is an extreme example, the number of tagging sequences with a low (<5) minimal Hamming distance suggests that extra correction may be required.

*Step 3 – Determining the cut-off for correction*: If extra correction is required, the dataset and the application may indicate which cut-off is most suitable. The increased number of tagging sequences associated with highly expressed targets is expected to result in lower minimal Hamming distances. The distribution of minimal Hamming distances of tagging sequences with one or few reads, can help decide on where to place the cut-off. When considering a higher cut-off (3 or more), it may be worthwhile to evaluate whether correction of the higher Hamming distances is concentrated in either the barcode or the UMI. If only the barcode or only the UMI is being corrected, it may indicate, respectively, that two transcripts in two different cells with a similar barcode have the same UMI or that two transcripts in the same cell have a similar UMI. These two scenarios become more likely for highly expressed transcripts.


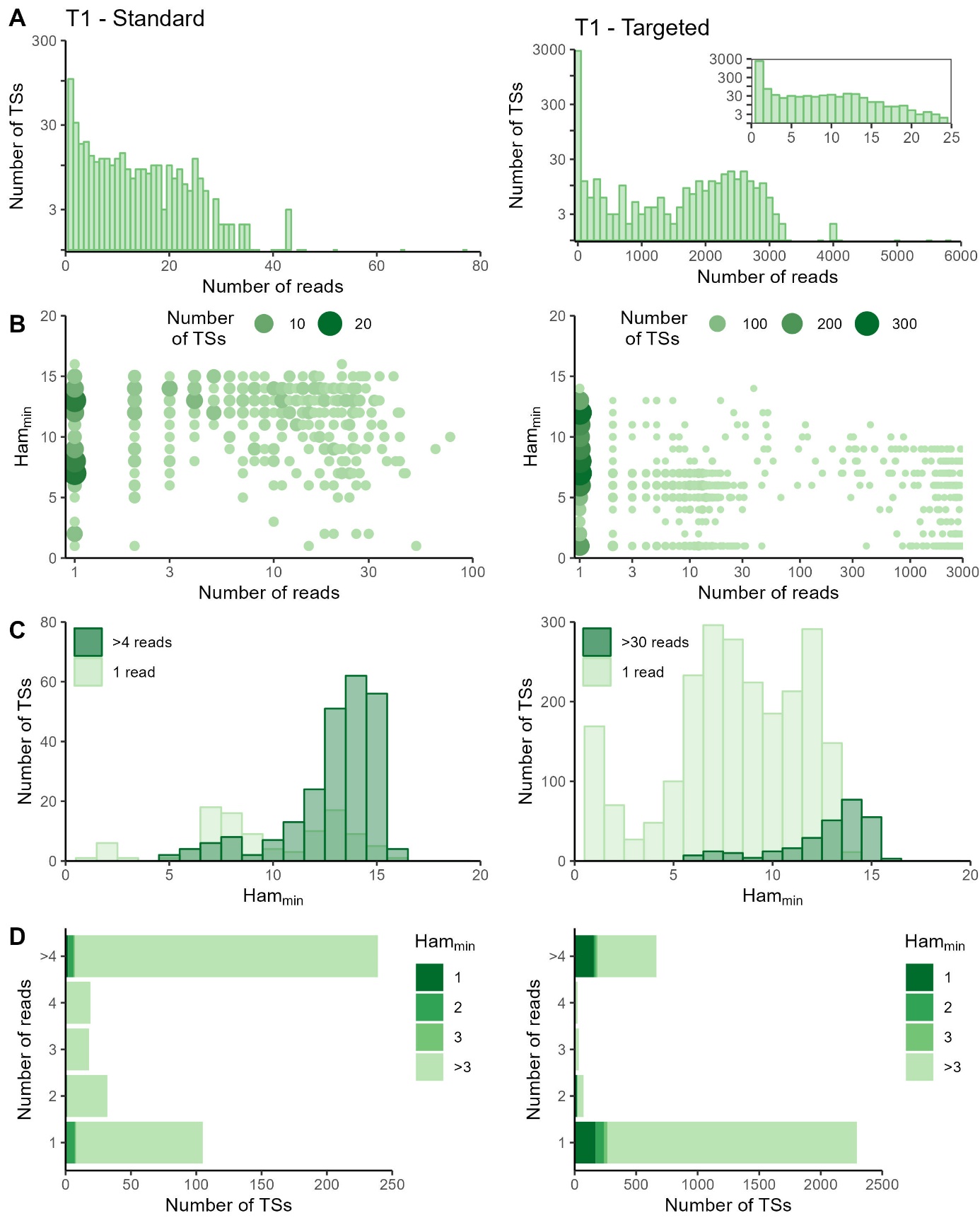
Example 2 – TREX1 T1 targeted: the plot on the right suggests that a cut-off of 3 may be suitable, as there is a local minimum at Ham_min_ is 3 for the tagging sequences covered by 1 read.
