# Supplementary material for: A targeted sequencing extension for transcript genotyping in single-cell transcriptomics
Source: Life Sci Alliance. 2023 Sep 11;6(11):e202301971. doi: 10.26508/lsa.202301971 (PMC10494938; doi:10.26508/lsa.202301971)
Supplement: Supplemental Data 2. — Creating test cDNA for primer optimization. [file LSA-2023-01971_Supplemental_Data_2.docx]

**Note S2 – Creating test cDNA for primer optimization**

Sample selection: Select RNA samples of sufficiently high concentration (>150 ng/µL) that were extracted from a subset of cells as close as possible to the sample you plan on using for scRNAseq to obtain the most representative results.

Protocol – 3’ method: Protocol is optimized for Chromium Single Cell Gene Expression Solution (v3 and v3.1, single index). We recommend including a no template control and other standard controls to evaluate success and contamination.

1. Thaw RNA and dilute with RNase free water to a concentration of 150 ng/µl (1 µl needed).
2. Add 2 µl of 3’ poly(dT) (100 µM) to 1 µl diluted RNA.
3. Reverse transcription:
   1. Make the RT mix. Add the RNA – 3’ poly(dT) mixture last.

| Component | Volume for 1 reaction (µl) |
| --- | --- |
| H2O (RNase free) | 2.75 |
| 5X Maxima RT buffer | 2 |
| 10 mM dNTPs | 1 |
| RNase inhibitor | 0.25 |
| 100 µM TSO | 0.5 |
| Maxima H minus RTase | 0.5 |
| RNA – 3’ poly(dT) mix | 3 |
|  | 10 |

- 1. Incubate the RT mix in a thermal cycler for 30 minutes at 50 °C, followed by 5 minutes at 85 °C.

1. ExoSap-IT cleanup:
   1. Add 2 µl ExoSap-IT to 10 µl cDNA.
   2. Incubate in thermal cycler for 15 minutes at 37 °C, followed by 15 minutes at 80 °C.
2. cDNA amplification:
   1. Make PCR mix. Add the cDNA last.

| Component | Volume (µl) |
| --- | --- |
| 10 µM forward primer | 0.75 |
| 10 µM reverse primer | 0.75 |
| 2X KAPA HiFi Hotstart ReadyMix | 12.5 µl |
| Cleaned-up cDNA | 12 |
|  | 26 |

- 1. Incubate in thermal cycler, using the following scheme.

| Temperature (°C) | Time |  |
| --- | --- | --- |
| 95 | 3’ |  |
| 98 | 20” |  |
| 65 | 15” | 25 cycles |
| 72 | 3’ |  |
| 72 | 5’ |  |
| 4 | ∞ |  |

1. SPRI cleanup
   1. Remove primer dimers with left-sided clean-up (ratio 1.0x) – see [USER GUIDE](https://research.fredhutch.org/content/dam/stripe/hahn/methods/mol_biol/SPRIselect%20User%20Guide.pdf) for protocol.
2. Measure concentration with Qubit and/or measure fragment length distribution with TapeStation, BioAnalyzer or similar.

An example of a successful round of creating 3’ test cDNA:

- Qubit dsDNA high sensitivity (HS) kit: 108 ng/µl
- Bioanalyzer:

Protocol – 5’ method: Protocol is optimized for Chromium Single Cell Immune Profiling Solution (5’, v1.1, single index). We recommend including a no template control and other standard controls to evaluate success and contamination.

1. Thaw RNA and dilute with RNase free water to a concentration of 150 ng/µl (1 µl needed).
2. Add 2 µl of 5’ poly(dT)(100 µM) to 1 µl diluted RNA.
3. Reverse transcription:
   1. Make the RT mix. Add the RNA –5’ poly(dT) mix last.

| Component | Volume for 1 reaction (µl) |
| --- | --- |
| H2O (RNase free) | 2.75 |
| 5X Maxima RT buffer | 2 |
| 10 mM dNTPs | 1 |
| Rnase inhibitor | 0.25 |
| 100 µM TSO | 0.5 |
| Maxima H minus RTase | 0.5 |
| RNA – 5’ poly(dT)mix | 3 |
|  | 10 |

- 1. Incubate the RT mix in a thermal cycler for 30 minutes at 50 °C, followed by 5 minutes at 85 °C.

1. ExoSap-IT cleanup:
   1. Add 2 µl ExoSap-IT to 10 µl cDNA.
   2. Incubate in thermal cycler for 15 minutes at 37 °C, followed by 15 minutes at 80 °C.
2. cDNA amplification:
   1. Make PCR mix. Add the cDNA last.

| Component | Volume (µl) |
| --- | --- |
| 10 µM forward primer | 0.75 |
| 10 µM reverse primer | 0.75 |
| 2X KAPA HiFi Hotstart ReadyMix | 12.5 µl |
| Cleaned-up cDNA | 12 |
|  | 26 |

- 1. Incubate in thermal cycler, using the following scheme.

| Temperature (°C) | Time |  |
| --- | --- | --- |
| 95 | 3’ |  |
| 98 | 20” |  |
| 65 | 15” | 25 cycles |
| 72 | 3’ |  |
| 72 | 5’ |  |
| 4 | ∞ |  |

1. SPRI cleanup
   1. Remove primer dimers with left-sided clean-up (ratio 1.0x) – see [USER GUIDE](https://research.fredhutch.org/content/dam/stripe/hahn/methods/mol_biol/SPRIselect%20User%20Guide.pdf) for protocol.
2. Measure concentration with Qubit and/or measure fragment length distribution with TapeStation, BioAnalyzer or similar.

An example of a successful round of creating 5’ test cDNA:

- Qubit dsDNA high sensitivity (HS) kit: 95.4 ng/µl
- Bioanalyzer:
