# Supplementary material for: A targeted sequencing extension for transcript genotyping in single-cell transcriptomics
Source: Life Sci Alliance. 2023 Sep 11;6(11):e202301971. doi: 10.26508/lsa.202301971 (PMC10494938; doi:10.26508/lsa.202301971)
Supplement: Supplemental Data 3. — Primer design and optimization and choosing technical parameters. [file LSA-2023-01971_Supplemental_Data_3.docx]

**Note S3 – Primer design and optimization and choosing technical parameters**

Primer design:

1. Determine which transcripts or transcript regions you want to design primers for. Also choose whether you want to use 3’ or 5’ chemistry. Depending on this choice, select the appropriate template below.
2. Download transcript sequences of genes of interest (e.g. from Ensembl genome browser) and paste the 5’ to 3’ transcript sequence (cDNA) in the template. Highlight region(s) of interest in the transcript.

If alternate transcripts are available, the nature and context of the application should indicate how to deal with them. Possible ways to handle them include focusing on the most common transcript(s), working with parts of the sequences shared between alternate transcripts or designing multiple primers each focusing on a transcript.

1. Only for 3’: take the reverse complement of the sequence in cell 2. Indicate the region of interest within the reverse complement.

Multiple online tools are available to facilitate generation of the reverse complement. We frequently use <https://www.bioinformatics.org/sms/rev_comp.html>.

1. Design a reverse primer located between 20 and 90 bp after the location of interest. Use “CTACACGACGCTCTTCCGATCT” as forward primer (and add this sequence to the start of the template).

We designed primers with Primer-BLAST (<https://www.ncbi.nlm.nih.gov/tools/primer-blast/index.cgi?GROUP_TARGET=on>).

| **3’ method** | **Instructions** | **Sequences** |
| --- | --- | --- |
| Step 1 | Choose a clear name. |  |
| Step 2 | Paste the 5’ to 3’ transcript sequence (cDNA).  Indicate the region of interest. |  |
| Step 3 | Take the reverse complement of the sequence in cell 2.  Indicate the region of interest. |  |
| Step 4 | Design a reverse primer located between 20 and 90 bp after the location of interest.  Use “CTACACGACGCTCTTCCGATCT” as forward primer (and add this sequence to the start of the template).  Color the location of the primer yellow in the sequence in cell 3. |  |
| Step 5 | Replace “NAME” by the name given in cell 1.  Add the primer from cell 4 behind the sequence of the tail. | PCR1_rv_NAME_3’:  GTGACTGGAGTTCAGACGTGTGCTCTTCCGATCT |

| **5’ method** | **Instructions** | **Sequences** |
| --- | --- | --- |
| Step 1 | Choose a clear name. |  |
| Step 2 | Paste the 5’ to 3’ sequence (cDNA). Indicate the region of interest. |  |
| Step 4 | Design a reverse primer located between 20 and 90 bp after the location of interest. Use “CTACACGACGCTCTTCCGATCT” as forward primer (and add this sequence to the start of the template).  Color the location of the primer yellow in the sequence in cell 2. |  |
| Step 5 | Replace “NAME” by the name given in cell 1.  Add the primer from cell 3 behind the sequence of the tail. | PCR1_rv_NAME_5’:  GTGACTGGAGTTCAGACGTGTGCTCTTCCGATCT |

Primer testing:

1. Prepare the following PCR mix – adjust the amounts as necessary for testing primers in parallel or at a temperature gradient.

| **Reagent** | **Volume (µl)** |
| --- | --- |
| Water | 9 |
| 2X KAPA HiFi HotStart ReadyMix | 12.5 |
| 10 µM PCR1_fw primer | 0.75 |
| 10 µM transcript-specific PCR1_rv primer | 0.75 |
| 3’ or 5’ test cDNA | 2 |

1. Incubate in a thermal cycler with the following settings:

| Temperature (°C) | Time | Repetition |
| --- | --- | --- |
| 95°C | 3’ |  |
| 98°C | 20” | 30 cycles |
| 65°C (or other or gradient) | 15” |  |
| 72°C | 30” |  |
| 72°C | 1’ |  |

1. Remove remaining primers with SPRI (1.0x or adapted depending on expected fragment length).
2. Assess fragment sizes in the PCR product using BioAnalyzer (Agilent), TapeStation (Agilent) or similar.

Example of successful reaction:

1. If a fragment of the expected length is detected (or multiple lengths depending on alternative transcripts), confirm that the correct transcript is targeted with Sanger sequencing. A quick method to confirm targeting of the correct transcript is to BLAST the automatically generated txt output and check whether the targeted transcripts is a top hit. This does not guarantee that the correct part of the transcript is targeted. In addition, it will not work in case of multiple alternative transcripts (very noisy data expected). In that scenario, look at the raw data and try to find the expected sequences manually.
2. If primers work individually, a similar setup can be used to test them in multiplex.

To consider when testing a multiplex reaction:

- The reverse primers should each be added with the concentration mentioned above.
- Sanger sequencing should be done with one reverse primer at a time. This may require scaling up the volumes in the PCR reaction.

Choosing technical parameters:

Based on our systematic analyses, we can advise the following for choosing amount of input cDNA, number of PCR cycles and sequencing depth.

- Amount of input cDNA: More input cDNA increases the number of transcripts that can be detected and results in less artificial increase in number of detected transcripts. Our general recommendation is thus to use as much input as possible. However, depending on the context, one can opt to include only part of the amplified cDNA, giving the opportunity to create additional targeted libraries later on, potentially with different or more targets.
- Number of PCR cycles: Our data indicates that 20 PCR cycles (both in PCR1 and PCR2) results in more transcripts being detected and less artificial increase. We have not tested it, but increasing the number of cycles may further improve results. We do believe that the increase in transcripts will be capped, with increases in number of cycles above an unknown threshold not resulting in the detection of new transcripts.
- Sequencing depth: Increased sequencing depth increases the number of detected transcripts, but also results into more artificial increase. Two factors should be taken into account when deciding on sequencing depth. First, the artificial increase can be addressed by adding the correction step based on Hamming distance. Second, additional sequencing can be done if the leftover targeted library is stored appropriately (-20°C for short term, -80°C for long-term).

While these recommendations can help with deciding the range for the technical parameters, we recommend to further optimize, especially if samples are valuable.
