# Supplementary material for: A targeted sequencing extension for transcript genotyping in single-cell transcriptomics
Source: Life Sci Alliance. 2023 Sep 11;6(11):e202301971. doi: 10.26508/lsa.202301971 (PMC10494938; doi:10.26508/lsa.202301971)
Supplement: Supplemental Data 4. — Code snippets. [file LSA-2023-01971_Supplemental_Data_4.docx]

***Note S4 – Code snippets***

*Extract tagging sequences of transcripts of interest from BAM file (in UNIX environment)*

With the following command, the reads in the BAM file mapped to the genomic region of interest are filtered for being annotated to the transcript of interest. For the filtered reads, the cellular barcode and the UMI (after CellRanger correction) are extracted and written to a file.

samtools view $BAM_file $genomic_region | grep $transcript_name | awk '{for(i=1;i<=NF;i++){if($i~/^CB:Z/){a=$i} if($i~/^UB:Z/){b=$i} print a,b}' > tagging_sequences.txt

*Further processing (in R)*

Load the required libraries.

library(data.table)

library(fread)

library(ggplot2)

library(stringr)

Plot distribution of number of reads per tagging sequence.

# Read in files with tagging sequences, count reads per tagging sequence and remove duplicate rows.

df <- fread("tagging_sequences.txt", header = FALSE)

df[, reads := .N, by = .(barcode, UMI)]

df <- distinct(df)

# Plot distribution of number of reads per tagging sequence.

ggplot() +

geom_histogram(data = df, aes(x = reads), binwidth = 1)

Extract genotype info from VarTrix debug file.

# Prepare data structure to save data.

df <- data.frame("index" = numeric(), "UMI" = character(),

"ref" = numeric(), "alt" = numeric(), "unk" = numeric())

# Read in file.

file_vartrix = "" # path to file

input <- readLines(con = file_vartrix)

# Extract data from file.

for (line in input) {

# Lines with info of interest contain "cell_index".

if (str_detect(line, pattern="cell_index")) {

split_line <- str_split(line, pattern = " ")[[1]]

index <- as.numeric(split_line[5])

UMI <- as.character(split_line[8])

ref <- as.numeric(split_line[12])

alt <- as.numeric(split_line[14])

unk <- as.numeric(split_line[16])

df <- rbind(df, list(index, UMI, ref, alt, unk))

}

}

# Add barcodes.

barcodes <- fread(file_barcodes, header=F)

barcodes$index <- 0:(nrow(barcodes)-1)

colnames(barcodes) <- c("barcode","index")

df <- merge(df, barcodes)

Calculate and plot minimal Hamming distance.

# Read in tagging sequences, calculate reads and concatenate BC and UMI.

df <- fread("tagging_sequences.txt", header = FALSE)

df[, reads := .N, by = .(barcode, UMI)]

df <- distinct(df)

df$tagseq <- paste0(df$barcode, df$UMI, sep = "_")

# # ONLY if restricting calculation to most abundant transcripts.

# df <- df[reads > 30, , ]

# Calculate minimal Hamming distance.

df$hamming <- 30

for(i in 1:(nrow(df)-1)) {

for (j in (i+1):nrow(df)) {

ham <- StrDist(df[i, tagseq], df[j, tagseq], method = "hamming")[1]

df[i, hamming := min(df[i, hamming], ham)]

df[j, hamming := min(df[j, hamming], ham)]

}

}

# Write minimal hamming distances to a file.

fwrite(df, "df_minimal_hamming.tab", sep = "\t")

# Count plot

ggplot(data = df, aes(x = reads, y = hamming)) +

geom_count(aes(colour = after_stat(n)))

# Histogram

ggplot() +

geom_histogram(data = df[reads == 1, , ], aes(x = hamming, fill = "red"), binwidth = 1, alpha = 0.4) +

geom_histogram(data = df_abundant, aes(x = hamming, fill = green), binwidth = 1, alpha = 0.4)

Correct tagging sequences based on Hamming distance.

# Read in tagging sequences, calculate reads, concatenate BC and UMI, calculate weight and number tagging sequences.

df <- fread("tagging_sequences.txt", header = FALSE)

df[, reads := .N, by = .(barcode, UMI)]

df <- distinct(df)

df$tagseq <- paste0(df$barcode, df$UMI, sep = "_")

df$weight <- df$reads/sum(df$reads)

df$number <- 1:nrows(df)

# Prepare data structure in which to save the corrected data.

df_corrected <- data.frame("barcode" = character(), "UMI" = character(), "reads" = numeric(), "tagseq" = character(), "weight" = numeric(), "number" = numeric(), "lead" = numeric(), "bc_support" = numeric(), "umi_support" = numeric(), "barcode_corr" = character(), "UMI_corrected" = character())

# Correct tagging sequences.

last <- nrow(df)

df_sub <- df[1, ]

while (1 <= last) {

df_sub <- df[1, ]

j <- 2

while (j <= last) {

if(StrDist(df[1, tagseq], df[j, tagseq], method = "hamming")[1] <= 3) {

df_sub <- rbind(df_sub, df[j, ])

}

j <- j+1

}

df_sub <- df_sub %>%

mutate(lead = min(number)) %>%

group_by(barcode) %>%

mutate(bc_support = sum(weight)) %>%

group_by(UMI) %>%

mutate(umi_support = sum(weight)) %>%

ungroup()

bc <- df_sub %>%

filter(bc_support = max(bc_support)) %>%

pull(barcode)

umi <- df_sub %>%

filter(umi_support = max(umi_support)) %>%

pull(UMI)

df_sub$barcode_corr <- bc

df_sub$UMI_corr <- umi

df_corrected <- rbind(df_corrected, df_sub)

df <- df %>%

filter(! number %in% (df_sub %>% pull(number)))

last <- nrow(df)

}

# Write corrected tagging sequences to file.

fwrite(df_corrected, "corrected_tagging_sequences.tab", sep = "\t")

Correct tagging sequences based on Hamming distance for two datasets simultaneously.

# Read in tagging sequences, calculate reads, concatenate BC and UMI, calculate weight and number tagging sequences.

df1 <- fread("tagging_sequences_1.txt", header = FALSE)

df1[, reads := .N, by = .(barcode, UMI)]

df1 <- distinct(df1)

df1$tagseq <- paste0(df1$barcode, df1$UMI, sep = "_")

df1$dataset <- "df1"

df1$weight <- df1$reads/sum(df1$reads)

df2 <- fread("tagging_sequences_2.txt", header = FALSE)

df2[, reads := .N, by = .(barcode, UMI)]

df2 <- distinct(df2)

df2$tagseq <- paste0(df2$barcode, df2$UMI, sep = "_")

df2$dataset <- "df2"

df2$weight <- df2$reads/sum(df2$reads)

df <- rbind(df1, df2) %>%

arrange(-weight) %>%

mutate(number = 1:n())

# Prepare data structure in which to save the corrected data.

df_corrected <- data.frame("barcode" = character(), "UMI" = character(), "reads" = numeric(), "tagseq" = character(), "dataset" = character(), "weight" = numeric(), "number" = numeric(), "lead" = numeric(), "bc_support" = numeric(), "umi_support" = numeric(), "barcode_corr" = character(), "UMI_corrected" = character())

# Correct tagging sequences.

last <- nrow(df)

df_sub <- df[1, ]

while (1 <= last) {

df_sub <- df[1, ]

j <- 2

while (j <= last) {

if(StrDist(df[1, tagseq], df[j, tagseq], method = "hamming")[1] <= 3) {

df_sub <- rbind(df_sub, df[j, ])

}

j <- j+1

}

df_sub <- df_sub %>%

mutate(lead = min(number)) %>%

group_by(barcode) %>%

mutate(bc_support = sum(weight)) %>%

group_by(UMI) %>%

mutate(umi_support = sum(weight)) %>%

ungroup()

bc <- df_sub %>%

filter(bc_support = max(bc_support)) %>%

pull(barcode)

umi <- df_sub %>%

filter(umi_support = max(umi_support)) %>%

pull(UMI)

df_sub$barcode_corr <- bc

df_sub$UMI_corr <- umi

df_corrected <- rbind(df_corrected, df_sub)

df <- df %>%

filter(! number %in% (df_sub %>% pull(number)))

last <- nrow(df)

}

# Write corrected tagging sequences and dataset info to file.

fwrite(df_corrected, "corrected_tagging_sequences.tab", sep = "\t")
